# Congenital anomalies in Okara District of Pakistan: Epidemiology, spectrum and ethno-demographic inequalities

**DOI:** 10.12669/pjms.41.3.9574

**Published:** 2025-03

**Authors:** Aqeela Nawaz, Ayesha Siddiqui, Mahnoor Mughal, Saima Naz, Muhammad Wajid, Sajid Malik

**Affiliations:** 1 Aqeela Nawaz Department of Zoology, Faculty of Life Sciences, University of Okara, Pakistan; 2 Ayesha Siddiqui Human Genetics Program, Department of Zoology, Quaid-i-Azam University, Islamabad, Pakistan; 3 Mahnoor Mughal Human Genetics Program, Department of Zoology, Quaid-i-Azam University, Islamabad, Pakistan; 4 Saima Naz Department of Anatomy, Rawalpindi Medical University, Rawalpindi, Pakistan; 5 Muhammad Wajid Department of Zoology, Faculty of Life Sciences, University of Okara, Pakistan; 6 Sajid Malik Human Genetics Program, Department of Zoology, Quaid-i-Azam University, Islamabad, Pakistan

**Keywords:** Birth defects, Epidemiology, Limb defects, Neurological defects, Descriptive genetics

## Abstract

**Objectives:**

To investigate the epidemiology, spectrum and ethno-demographic attributes of congenital anomalies (CA) in Okara district of Pakistan.

**Methods:**

In this cross-sectional study, individuals affected by CA were identified through door-to-door visits, surveys of public places, and district hospitals during the period 2015 to 2022 in Okara district of Punjab, Pakistan. Descriptive statistics were employed, and CA were classified using the International Classification of Diseases (ICD-10) and Online Mendelian Inheritance in Man (OMIM) databases.

**Results:**

A total of 709 individuals/families with certain types of CA were ascertained. These anomalies were categorized into eight major and 95 minor entities. Limb defects predominated among the major groups (21%), followed by neurological disorders (16%), neuromuscular disorders (15%), sensorineural/ear defects (15%), ectodermal defects (9%), musculoskeletal defects (7%), and others (4%). Parental consanguinity was observed in 67% of the cases and 55% anomalies had familial occurrence. There were statistically significant differences among the major categories with respect to rate of parental consanguinity and familial/sporadic nature. Marked disparities were also observed in the distribution of CA with respect to ethnic groups, economic quartiles and age categories of index cases.

**Conclusions:**

Neurological and neuromuscular defects which accounted for 31% of the sample, were of severe nature and cause of disability, whereas limb defects were mainly of minor nature. There is wide variation in the prevalence of CA across the ethno-demographic variables. A multifaceted strategy that explicitly includes marginalized and minority populations in health services and addresses environmental, genetic, and maternal health factors is necessary to prevent CA.

## INTRODUCTION

Congenital anomalies (CA) include a spectrum of anatomical, physiological, and metabolic abnormalities that occur during development and manifest prenatally, perinatally, or postnatally.^1^ As infectious diseases are better controlled and nutrition, healthcare and hygiene have improved, consequently, CA have become the leading cause of morbidity and mortality. Although CA are individually rare, their combined impact is nevertheless substantial.^2^

An estimated 6% of babies worldwide are born with a CA.^1^ CA are the cause of mortality of 240,000 newborns within four weeks of birth every year, and cause a further 170,000 deaths of children between the ages of one month and five years.^1^,^2^ The burden of CA in Pakistan is tremendous due to several factors, including the high rate of consanguineous marriages, higher parity, low socioeconomic conditions, lack of prenatal care, and poor health facilities in rural areas.s^3^-^4^ In a study carried out in the southern coastal city of Karachi, CA had a frequency of 11.4 per 1,000 live births, highlighting neural tube defects as the most prevalent anomalies.^5^

In Nawabshah district of Sindh, Pakistan, Arijo et al.^6^ witnessed that 12% of neonates had birth defects comprising of congenital hydrocele, spina bifida, cleft lip/palate and club foot, and remarked that CA were associated with lack of folic acid use during pregnancy and cousin marriages. In recent study carried out in Karachi, Pakistan, Anbreen et al.^7^ observed that the prevalence of CA was 2.2% and most commonly identified abnormality was of central nervous system 60%, followed by renal 12%, gastrointestinal 5% and others 18%. In a study conducted in Rawalpindi Medical University Allied hospitals, Rawalpindi, Umer et al.^8^ revealed that among Pakistani infants, the prevalence of congenital heart defects (CHD) was found to be 4 per 1000 live births.^8^ Another study carried out on the Pakistani immigrants in the UK revealed that congenital heart defects were among the most common CA with a prevalence of 11 per 1,000 live births, which was significantly higher compared to non-Asian populations.^9^ An estimated 9% of perinatal deaths in Pakistan are due to CA.^10^-^11^

Ninety percent of infants with serious CA are born in low- and middle-income nations. In Pakistan, the rural population is in majority and the rural areas are with inadequate healthcare facilities.^12^ Hence, CA poses an additional burden on Pakistan’s already strained healthcare system. Epidemiological studies and impact assessment are essential for identifying priority areas to ensure optimal utilization of resources.

Okara district is situated in the eastern part of the central Punjab, Pakistan. In Okara district, the prevalence of low-birth-weight babies was 4%, and neonatal death in the health facility was 4%.^13^ Data from the Multiple Indicator Cluster Survey (MICS) 2017-2018 and the Pakistan Social and Living Standards Measurement (PSLM) 2020 revealed that Okara exhibits a higher unmet need for family planning services (72%) compared to the provincial average (66%), indicating challenges in reproductive health services.^14^

In the context of maternal health, 73% of deliveries in Okara occur in health facilities, slightly lower than the Punjab average of 80%. Additionally, 70% of mothers receive postnatal health checks, compared to the provincial average of 80%, highlighting areas for improvement in maternal healthcare services. Like many other small towns of Pakistan, not much is known about the prevalence of CA in Okara district. Previously, Sadiq et al. reported that among singleton pregnancies complicated with polyhydramnios in Okara district, 16% of pregnancies resulted in CA in the infant.^15^ Khalid et al. observed that mothers with non-communicable diseases who delivered at District Head Quarter Hospital, Okara, had 59% of infants with CA.^16^ However, the epidemiology of CA in the general population of Okara is not known. To this end, the present study was carried out to draw a comprehensive picture of the spectrum of CA in Okara district.

## METHODS

A clinical and epidemiological study was carried out from 2015 to 2022 in Okara district of Punjab, Pakistan (www.pbs.gov.pk/). In a cross-sectional study design, patients with CA were included from district headquarters hospitals and special education centers. Patients were also included through random door-to-door visits and convenience sampling in educational institutes and community centers. The index cases were brought to the nearest health facilities for medical examination and diagnosis. The data on phenotypic and bio-demographic variables and pedigree information were collected on a structured proforma.

### Inclusion & Exclusion Criteria:

The participants were included irrespective of their gender, ethnicity, origin and type of anomalies. Only the individuals with malformations of congenital and/or hereditary nature were included. Patients presented with anomalies with likely infectious nature or traumatic conditions were excluded. The patients/families providing incomplete information were not included. The sample size was calculated through sample size formula for proportion estimation.

### Ethical Approval:

The Ethical Review Committee of Quaid-i-Azam University, Islamabad, granted approval for carrying out this study (DAS-19; Dated: July 3, 2019). Ethical approvals were also obtained from DHQ Hospital, Okara (No. 6516-18) and THQ Hospital, Depalpur (No. 186-89). After receiving verbal consent of the participants or their parents/guardians, the data were collected according to the Helsinki Declaration. All the data were acquired and documented in the presence of the family head or guardian after participants had provided informed written or formally documented verbal consent when illiterate. We followed the cross-sectional reporting guidelines provided by the Strengthening the Reporting of Observational Studies in Epidemiology (STROBE).^17^

### Classification of anomalies and statistical analysis:

Index cases underwent medical examination by the specialized medical doctors. Patients from distant locations were brought to the nearest hospital for diagnosis. Definition of CA was established using a standardized coding system following OMIM (https://www.omim.org/) and ICD-10 (https://icd.who.int/browse10/2019/en) databases. The CA were divided into major categories based on the involvement of respective organ-systems, i.e., neurological disorders, neuromuscular disorders, musculoskeletal defects, visual impairments, sensorineural anomalies, limb defects, and metabolic disorders. For intellectual disabilities, the DSM-5 criteria were utilized for the sub-categorization.^18^ For limb defects, we utilized the criteria of Temtamy and McKusick.^19^ Descriptive summaries were generated through MS-Excel and STATA, and the significance of the distribution was checked through chi-square test statistics. Proportions and corresponding 95% confidence intervals (CI) were calculated for the CA.

## RESULTS

### Sample characteristics and classification of congenital anomalies:

A total of 709 index cases (467 males, 242 females) from independent families with certain types of CA were recruited. The representation of individuals from rural areas was 49%, majority spoke Punjabi (95%), and 43% belonged to poor socio-economic quartile. The CA were categorized into eight major and at least 95 minor entities (Table-I, II). Among the major categories, limb defects were the most frequent (n=145; 21%), followed by neurological disorders (n=116; 16%), neuromuscular anomalies (n=109; 15%), sensorineural/ear defects (n=108; 15%), eye/visual impairments (n=96; 14%), ectodermal defects (n=64; 9%), musculoskeletal defects (n=46; 7%), and others (n=25; 4%) (Table-I).

**Table-I:** Major categories of congenital anomalies.

Major category	Index cases, n (%)*			Proportion	95% CI

	Male	Female	Total		
Limb defects	100 (69)	45 (31)	145	0.205	0.175-0.234
Neurological disorders	79 (68)	37 (32)	116	0.164	0.136-0.191
Neuromuscular anomalies	79 (72)	30 (28)	109	0.154	0.127-0.180
Sensorineural/ear defects	66 (61)	42 (39)	108	0.152	0.126-0.179
Eye/visual impairments	60 (63)	36 (38)	96	0.135	0.110-0.161
Ectodermal defects	37 (58)	27 (42)	64	0.090	0.069-0.111
Musculoskeletal defects	30 (65)	16 (35)	46	0.065	0.047-0.083
Others	16 (64)	9 (36)	25	0.035	0.022-0.049
Total	467 (66)	242 (34)	709	1.000	--

* P=0.487.

**Table-II:** Major and minor categories of congenital anomalies.

Major/minor categories	Frequency	Proportion	95% CI	ICD-10	OMIM
***Limb defects***	***145***	***0.205***	***0.175-0.234***		
Polydactyly types	32	0.045	0.030-0.060	Q69.9	174200, 174400
Polydactyly, preaxial	27	0.038	0.240-0.052	Q69.1	174400
Talipes types	24	0.034	0.021-0.047	Q66.0	119800
Polydactyly, postaxial	14	0.020	0.009-0.030	Q69	174200
Brachydactyly	7	0.010	0.003-0.017	Q68.81	113000
Camptodactyly	6	0.008	0.002-0.015	Q74.0	114200
Amputations	5	0.018	0.008-0.028	Q73.8	217100
Syndactyly types	5	0.018	0.008-0.028	Q70	609815
Finger clubbing	4	0.006	0.000-0.011	R68.3	119900
Synpolydactyly	4	0.006	0.000-0.011	Q70.4	186000
Syndactyly I-a	3	0.004	-0.001-0.009	Q70.3	609815
Syndactyly I-c	3	0.004	-0.001-0.009	Q70.1	
Constriction band	3	0.004	-0.001-0.009	Q79.8	217100
Symbrachydactyly	2	0.003	-0.001-0.007	Q71.8	
Thumb hypoplasia	2	0.003	-0.001-0.007	Q71.3	188100
Clinodactyly	1	0.001	-0.001-0.004	Q74.0	148520
Finger contractures	1	0.001	-0.001-0.004	M72.0	
Radial hemimelia	1	0.001	-0.001-0.004	Q73.8	
Split-hand-foot	1	0.001	-0.001-0.004	Q72.7	183600
***Neurological disorders***	***116***	***0.164***	***0.136-0.191***		
Intellectual disability (ID) types	27	0.038	0.024-0.052	F70-79	
ID, mild	25	0.035	0.022-0.048	F70	249500
ID, moderate	21	0.029	0.017-0.042	F71	
Microcephaly	14	0.020	0.010-0.030	Q02	251200
ID, severe	9	0.013	0.004-0.021	F72, F73	611091
Macrocephaly	4	0.006	0.000-0.011	Q75.3	153470
Down syndrome	3	0.004	-0.001-0.009	Q90	190685
Epilepsy, pediatric seizures	3	0.004	-0.001-0.009		121200
Essential tremor	3	0.004	-0.001-0.009	G25	190300
Developmental delay	2	0.003	-0.001-0.007	Z13.42	618330
Epilepsy	2	0.003	-0.001-0.007	G40	117100
Spina bifida	2	0.003	-0.001-0.007	Q05	182940
Holoprosencephaly	1	0.001	-0.001-0.004	Q04.2	610829
***Neuromuscular anomalies***	***109***	***0.154***	***0.127-0.180***		
Cerebral palsy types	86	0.121	0.097-0.145	G80	605388
Muscular dystrophy	11	0.016	0.006-0.025	G71.0	310200
Muscle hypotonia (legs)	9	0.013	0.004-0.021	P94.2	300868
Amyotrophic lateral sclerosis	2	0.003	-0.001-0.007	G12.2	105400
Spinal muscular atrophy	1	0.001	-0.001-0.004	G12.1	253300
***Sensorineural/ear defects***	***108***	***0.152***	***0.126-0.179***		
Deaf-muteness	101	0.142	0.117-0.168	H91.3	304500
Mute only	3	0.004	-0.001-0.009	R47.0	
Microtia	2	0.003	-0.001-0.007	Q17.2	600674
Stuttering	2	0.003	-0.001-0.007	F98.5	184450
***Eye/visual impairments***	***96***	***0.135***	***0.110-0.161***		
Blindness	46	0.065	0.047-0.083	H54	216900
Squint eye	18	0.025	0.014-0.037	H50.9	185100
Night blindness	10	0.014	0.005-0.023	H53.60	310500
Retinitis pigmentosa	8	0.011	0.004-0.019	H35.5	603937
High myopia	5	0.007	0.001-0.013	H52.10	601075
Day blindness	3	0.004	-0.001-0.009	H53.1	
Nystagmus	2	0.003	-0.001-0.007	H55	617297
Anophthalmia	1	0.001	-0.001-0.004	Q11.2	251600
Cataract	1	0.001	-0.001-0.004	Q12.0	115700
Color blindness	1	0.001	-0.001-0.004	H53.5	303800
Glaucoma	1	0.001	-0.001-0.004	Q15.0	231300
***Ectodermal defects***	***64***	***0.090***	***0.069-0.111***		
Albinism	9	0.013	0.004-0.021	E70.3	203100
Dermatitis	9	0.013	0.004-0.021	L20	603165
Ichthyosis	8	0.011	0.004-0.019	Q80	242300
Vitiligo	8	0.011	0.004-0.019	L80	606579
Alopecia areata	5	0.007	0.001-0.013	L63	104000
Psoriasis	4	0.006	0.000-0.011	L40	177900
Alopecia universalis	3	0.004	-0.001-0.009	L63.1	203655
Anodontia	2	0.003	-0.001-0.007	K00.0	206780
Hypodontia	2	0.003	-0.001-0.007	K00.0	604625
Keratoderma	2	0.003	-0.001-0.007	L86	144200
Koilonychia	2	0.003	-0.001-0.007	Q84.6	149300
Acral keratoderma caries syndrome	1	0.001	-0.001-0.004		607656
Auricular hypertrichosis	1	0.001	-0.001-0.004	Q84.2	307150
Dentinogenesis imperfecta	1	0.001	-0.001-0.004	K00.5	125490
Fluorosis	1	0.001	-0.001-0.004	K00.3	
Hereditary gingival fibromatosis	1	0.001	-0.001-0.004	K06.1	135300
Microdontia	1	0.001	-0.001-0.004	K00.2	
Nail/tooth atrophy	1	0.001	-0.001-0.004		
Nail atrophy	1	0.001	-0.001-0.004	L60.3	161050
Neurofibromatosis	1	0.001	-0.001-0.004	Q85.0	162200
Wooly hair	1	0.001	-0.001-0.004	Q84.1	616760
***Musculoskeletal defects***	***46***	***0.065***	***0.047-0.083***		
Dwarfism types	18	0.025	0.014-0.037	E34.3	100800
Skeletal dysplasia	7	0.010	0.003-0.017	Q79	618870
Arthrogryposis	5	0.007	0.001-0.013	Q74.3	108120
Osteoarthritis	4	0.006	0.000-0.011	M15.9	165720
Achondroplasia	3	0.004	-0.001-0.009	Q77.4	100800
Kyphoscoliosis	2	0.003	-0.001-0.007	M40	610170
Leg length discrepancy	2	0.003	-0.001-0.007	M21.7	
Apert syndrome	1	0.001	-0.001-0.004	Q87.0	101200
Genu varum	1	0.001	-0.001-0.004	Q74.1	
Hypertrophic osteoarthropathy	1	0.001	-0.001-0.004	M89.4	614441
Pectus excavatum	1	0.001	-0.001-0.004	Q67.6	169300
Rickets	1	0.001	-0.001-0.004	E34.4	100800
***Others***	***25***	***0.035***	***0.022-0.049***		
Thalassemia	7	0.010	0.003-0.017	D56	613985
Cleft-lip	3	0.004	-0.001-0.009	Q36	600625
Polycystic ovary syndrome	3	0.004	-0.001-0.009	E28.2	184700
Bardet-Biedl syndrome	2	0.003	-0.001-0.007	Q87.89	209900
Cleft-lip-palate	2	0.003	-0.001-0.007	Q37	119530
Hemophilia	2	0.003	-0.001-0.007	D66	306700
MCC deficiency	2	0.003	-0.001-0.007		609010
Undifferentiated genitalia	2	0.003	-0.001-0.007	Q56.4	250790
Progeria	1	0.001	-0.001-0.004	E34.8	176670
Wilson disease	1	0.001	-0.001-0.004	E83.01	277900

The limb defects were further divided into 19 subcategories. Among these, there was the highest representation of polydactylies (n=73), talipes (n=24), and syndactylies (n=15) (Table-II). Neurological disorders were grouped into 13 separate entities, intellectual disability (ID; n=82) and microcephaly (n=14) being more frequent. Neuromuscular anomalies were characterized into five sub-types, of which the most frequent were cerebral palsy types (n=86), followed by muscular dystrophy (n=11). Sensorineural/ear defects were resolved into four sub-types, eye/visual impairments into 11, ectodermal defects into 21, and musculoskeletal defects were divided into 12 sub-types.

### Consanguinity and familial/sporadic nature:

The parental consanguinity was 67% in the overall cohort. The highest rate of consanguinity was witnessed in sensorineural/ear defects (82%), followed by neurological disorders (75%), while the lowest was observed in limb and ectodermal defects (55% each; P<0.0001) (Table-III).

**Table-III:** Parental consanguinity and familial/sporadic nature of congenital anomalies.

Major category	Index cases	Parental consanguinity, n (%)*		Familial/sporadic nature, n (%)**	

		Present	Absent	Familial	Sporadic
Limb defects	145	80 (55)	65 (45)	78 (54)	67 (46)
Neurological disorders	116	87 (75)	29 (25)	53 (46)	63 (54)
Neuromuscular anomalies	109	77 (71)	32 (29)	46 (42)	63 (58)
Sensorineural/ear defects	108	89 (82)	19 (18)	68 (63)	40 (37)
Eye/visual impairments	96	65 (68)	31 (32)	58 (60)	38 (40)
Ectodermal defects	64	35 (55)	29 (45)	46 (72)	18 (28)
Musculoskeletal defects	46	28 (61)	18 (39)	26 (57)	20 (43)
Others	25	15 (60)	10 (40)	15 (60)	10 (40)
Total	709	476 (67)	233 (33)	390 (55)	319 (45)

* P<0.0001;

** P=0.002.

Pedigree structures of index cases showed that familial occurrence was 55% as compared to sporadic, 45% (Table-III). The familial cases were highest in ectodermal defects (72%) and sensorineural/ear defects (63%), whereas the highest ratio of sporadic cases was observed in neuromuscular anomalies (58%), followed by neurological disorders (54%), and the differences were statistically significant.

There were at least 110 cases of CA with a syndromic appearance. Intellectual disability was most commonly associated with deaf-muteness (n=10), eye/visual impairments with deaf-muteness (n=12), cerebral palsy (CP) with blindness (n=8), limb defects with high myopia (n=6), and musculoskeletal defects were associated with limb/digit defects (n=5).

### Ethnic and demographic differences in distribution of CA:

Disparities in the distribution of major categories of CA were evident in the age categories (Table-IV). There was highest representation of limb defects and eye/visual impairments in patients >29 years; whereas there was highest representation of cases with sensorineural/ear defects in >9-19 years category (P<0.0001). Further, statistically significant differences were observed in the distribution of major categories of CA with respect to economic quartiles (P<0.0001). Sensorineural/ear defects and neuromuscular anomalies were more prevalent among the subjects with poor and low-middle class families, whereas ectodermal and limb defects were more prevalent among the middle and high socio-economic class patients. Disparities in the distribution of major categories of CA were also evident among the ethnic groups; In Araien caste-system, neuromuscular anomalies and eye/visual impairments had the highest representation; in Rajpoot and Sheikh, limb defects had the highest representation; in Bhattis, neuromuscular anomalies had the highest representation; in Khokhars, Sensorineural/ear defects had the highest representation; and in Malik and Jutt, there was highest representation of ectodermal defects (P=0.0133).

**Table-IV:** Demographic and ethnic differentials in major categories of congenital anomalies.

Variables	Limb defects	Neurological disorders	Neuromuscular anomalies	Sensorineural/ ear defects	Eye/visual impairments	Ectodermal defects	Musculoskeletal defects	Others	Total, # (%)
Age categories (years)*									
≤ 9	31 (23)	29 (21)	24 (18)	8 (6)	14 (10)	12 (9)	5 (4)	13 (10)	136 (19)
>9-19	43 (16)	44 (16)	53 (20)	65 (24)	34 (13)	9 (3)	14 (5)	5 (2)	267 (37)
>19-29	30 (21)	26 (18)	21 (14)	19 (13)	15 (10)	21 (14)	9 (6)	5 (3)	146 (21)
>29	41 (26)	17 (11)	11 (7)	16 (10)	33 (21)	22 (14)	18 (11)	2 (1)	160 (23)
Total, # (%)	145 (20)	116 (16)	109 (15)	108 (15)	96 (14)	64 (9)	46 (6)	25 (4)	709 (100)
Economic quartile*									
Poor	51 (17)	48 (16)	53 (17)	58 (19)	39 (13)	23 (7)	28 (9)	8 (3)	308 (43)
Low	49 (23)	36 (17)	31 (15)	28 (13)	31 (15)	15 (7)	13 (6)	8 (4)	211 (30)
Middle	32 (24)	24 (18)	18 (14)	19 (14)	18 (14)	10 (8)	2 (2)	9 (7)	132 (19)
High	13 (22)	8 (14)	7 (12)	3 (5)	8 (14)	16 (28)	3 (5)	0	58 (8)
Caste system/ethnicity*									
Araien	21 (15)	23 (16)	25 (18)	20 (14)	26 (18)	12 (9)	4 (3)	10 (7)	141 (22)
Rajpoot	14 (24)	9 (15)	7 (12)	10 (17)	9 (15)	6 (10)	3 (5)	1 (2)	59 (9)
Bhatti	8 (15)	9 (16)	14 (25)	8 (15)	8 (15)	5 (9)	2 (4)	1 (2)	55 (8)
Sheikh	10 (37)	3 (11)	2 (7)	3 (11)	4 (15)	3 (11)	2 (7)	0	27 (4)
Khokhar	4 (15)	4 (15)	6 (23)	7 (27)	2 (8)	2 (8)	1 (4)	0	26 (4)
Malik	1 (4)	2 (8)	2 (8)	4 (15)	6 (23)	7 (27)	1 (4)	3 (12)	26 (4)
Jutt	6 (16)	4 (16)	4 (16)	1 (4)	3 (12)	6 (24)	3 (12)	0	25 (4)
Others	67 (23)	53 (18)	40 (14)	44 (15)	34 (12)	22 (8)	24 (8)	5 (2)	289 (45)

* P<0.0001; **P=0.0133.

## DISCUSSION

This study provides a detailed descriptive and clinical assessment of CA in the general population of Okara. A broad spectrum of anomalies was witnessed in this study. One of the key findings is that the limb defects were the most prevalent category in the present cohort of CA. These results align with other Pakistani studies and, hence, support the validity of both our study and the existing literature. In a study carried out in Sialkot, Pakistan, Bhatti et al. reported that limb defects were the most frequent (47%) category.^11^

In a large cohort of CA from the Hazara population of Pakistan, limb defects were the 2^nd^ largest group of CA, making up 25% of the cases.^3^ The independent studies carried out by Naeem et al. and Zahra et al. in north-western populations of Pakistan, limb defects were the 3^rd^ most common type.^4^,^20^ Similarly, a hospital-based study in Rawalpindi reported that limb defects were the 3^rd^ most common type.^21^ The prevalence of sub-types of limb defects, however, varied in these cohorts. This heterogeneity could be attributed to variation in genetic and non-genetic factors, socioeconomic and geographic factors, and diagnostic and reporting variations.^21^,^22^

Neurological disorders and neuromuscular anomalies are the leading causes of disability, and these categories were collectively present in 32% of current sample. In a study conducted in the Hazara population of Pakistan, ID types were the most prevalent abnormalities among neurological disorders.^3^ The same trend was observed in our cohort. Pakistan has one of the highest percentages of children with ID among developing countries.^23^ Many non-genetic factors such as low economic quantile, poor antenatal care, deprived health care facilities, advanced maternal age at birth, low parental education, rural origin, infections, maternal malnutrition, and exposure to teratogens contribute to the elevated rates of ID in developing countries, including Pakistan.^1^,^2^

Here, the most common neuromuscular defect was CP, which may result from various factors including preterm birth, placental abnormalities, birth asphyxia, intrauterine infection, and fetal growth restriction.^24^ Consanguinity has been shown to be associated with CA in many studies.^3^,^21^ In the present cohort, 67% cases had parental consanguinity. The highest consanguinity was observed in neuromuscular anomalies (82%), followed by sensorineural/ear defects (81%), and eye/visual impairments (68%). This level of consanguinity is much higher than the background consanguinity recorded in the Pakistani population in general as well as in the Okara population, which may suggest a high contribution of recessive genes in the etiology of these anomalies. In a study, Nawaz et al.^25^ observed that the rate of consanguinity in Okara district was 61%, with an estimated inbreeding coefficient of 0.036. Literature indicates that consanguinity significantly increases the risk for autosomal recessive disorders, particularly neurological disorders and sensorineural/ear defects.^10^,^15^,^21^

The distinction between sporadic and familial occurrence of CA is crucial for understanding their underlying causes, etiology, risk assessment, and planning appropriate preventive or management strategies. Familial cases may have a potential inherited genetic component and follow a specific inheritance pattern; on the other hand, sporadic cases may be attributed to new genetic mutations, environmental factors or stochastic developmental errors. In this cohort, sporadic occurrence of CA was more common compared to familial (55% vs. 45%; Table-II). The sporadic occurrence was more pronounced in ectodermal defects (72%), neurological disorders (63%), and eye/visual impairments (60%). In a sample of CA ascertained from north-western Pakistan, sporadic occurrence was evident in 76% cases.^4^ Sporadic occurrences was witnessed in 65% cases of CA ascertained from Hazara population of Pakistan.^3^ These differences may highlight etiological and genetic heterogeneity underlying CA in Pakistani communities. It is pertinent to mention that sporadic anomalies may occur mostly as a result of nongenetic causes such as environmental, nutritional, and maternal factors, infectious teratogenic agents during pregnancy, and physical factors such as high-energy radiation and hyperthermia.

This study further revealed that there were significant differences in the distribution of major categories of CA with respect to demographic and ethnic variables. This is the likely representation of differences in the parental consanguinity, genetic background, mutational burden and other etiological factors among these socio-demographic classes. It is important to recognize that preventing CA involves a multifaceted approach that addresses genetic, environmental, and maternal health factors. Adequate intake of folic acid and iodine through fortification of staple foods or supplementation, vaccination, regular prenatal care, and proactive health management are key components of preventing and managing potential risk factors. Pregnant women should be educated about the risks of exposure to teratogenic substances, including certain medications, alcohol, illicit drugs, tobacco, pesticides, pollutants, and radiation.

### Strengths and limitations:

This is the first comprehensive report on the descriptive epidemiology of CA in Okara district and it presents a detailed overview of major and minor anomalies prevalent in this population. This study has following limitations: first, it does not report true birth-prevalence of CA; second, it does not present the etiological factors underlying the CA; and third, the data collection was based on convenience sampling. Finally, the study results may not be applicable to the other populations.

## CONCLUSION

Current study is important as a part of community health surveillance of major CA that cause disabilities and a step towards the development of a national registry for CA in Pakistan. This is the first study which provides a detailed descriptive and clinical assessment of CA in the general population of Okara. Limb defects, neurological and neuromuscular anomalies, sensorineural/ear defects, and eye/visual impairments account for the highest burden (81% of the sample). In the backdrop of consanguinity, genetic counseling can help at-risk families to understand their specific genetic risks and options for early diagnosis and intervention. Furthermore, by integrating data on ethno-demographic disparities, counseling can be tailored to address the unique needs of diverse populations in the district, ensuring equity in healthcare access. It is the need of the hour to launch awareness campaigns and genetic testing programs for early detection and prevention. The health authorities can use these results to prioritize resource allocation for high-burden anomalies, such as neurological and neuromuscular defects.

### Authors’ Contribution:

**SM:** Literature search, Conceived, designed and supervised the study. Responsible and accountable for the accuracy and integrity of data.

**AN**, **AS**, **MM**, **SN** and **MW:** Literature search, Data collection and Statistical analysis. Critical Review.
